# A Multilevel Approach to Estimating Small Area Childhood Obesity Prevalence at the Census Block-Group Level

**DOI:** 10.5888/pcd10.120252

**Published:** 2013-05-02

**Authors:** Xingyou Zhang, Stephen Onufrak, James B. Holt, Janet B. Croft

**Affiliations:** Author Affiliations: Stephen Onufrak, James B. Holt, Janet B. Croft, Centers for Disease Control and Prevention, Atlanta, Georgia.

## Abstract

**Introduction:**

Traditional survey methods for obtaining nationwide small-area estimates (SAEs) of childhood obesity are costly. This study applied a geocoded national health survey in a multilevel modeling framework to estimate prevalence of childhood obesity at the census block-group level.

**Methods:**

We constructed a multilevel logistic regression model to evaluate the influence of individual demographic characteristics, zip code, county, and state on the childhood obesity measures from the 2007 National Survey of Children’s Health. The obesity risk for a child in each census block group was then estimated on the basis of this multilevel model. We compared direct survey and model-based SAEs to evaluate the model specification.

**Results:**

Multilevel models in this study explained about 60% of state-level variances associated with childhood obesity, 82.8% to 86.5% of county-level, and 93.1% of zip code-level. The 95% confidence intervals of block- group level SAEs have a wide range (0.795-20.0), a low median of 2.02, and a mean of 2.12. The model-based SAEs of childhood obesity prevalence ranged from 2.3% to 54.7% with a median of 16.0% at the block-group level.

**Conclusion:**

The geographic variances among census block groups, counties, and states demonstrate that locale may be as significant as individual characteristics such as race/ethnicity in the development of the childhood obesity epidemic. Our estimates provide data to identify priority areas for local health programs and to establish feasible local intervention goals. Model-based SAEs of population health outcomes could be a tool of public health assessment and surveillance.

## Introduction

The prevalence of childhood obesity tripled during the last 3 decades in the United States (1); data for 2009 through 2010, showed that 16.9% (approximately12.0 million) of US children aged 2 to 19 years were obese (2). Besides disparities in childhood obesity among various racial/ethnic groups (2–4), research shows significant disparities by geographic area: by state (5), city (6), and community (7). Small-area data can reveal wide disparities in obesity outcomes and facilitate community-based initiatives for obesity prevention (8). Having reliable data for each community or small area allows state, county, and local decision makers and health professionals to tailor programs for preventing childhood obesity to conditions and factors that affect their community (9), identify priority areas for action, and optimize the use of limited resources.

Local public health practitioners often lack small-area data on childhood obesity. National health surveys, such as the National Health and Nutrition Examination Survey (NHANES) (www.cdc.gov/NCHS/nhanes.htm), the National Survey of Children’s Health (NSCH) (www.cdc.gov/nchs/slaits/nsch.htm), and the Youth Risk Behavior Survey (YRBS) were designed to provide data on national or state childhood obesity. Direct estimates of obesity rates in small areas or communities cannot be calculated on the basis of data gathered through these surveys. Use of the surveillance methods for obtaining national (ie, large-area) data to obtain small-area data on childhood obesity is prohibitively expensive.

There are, however, cost-effective methods of generating health-related data, particularly on obesity, for small-area populations (10–12). Recently, considerable research has been done on multilevel, model-based, small-area estimation methods (10,13–15). These methods can produce data on variations in the multilevel influence of local social and physical environments on health outcomes among people in small areas by using various demographic characteristics (eg, age, sex, race/ethnicity). Another advantage is that model-based small-area estimation methods borrow information from both individual-level data within the survey sample and from area-level covariates external to the original sample, and they tend to generate smoothed estimates with better precision (16). Malec et al constructed a 2-stage hierarchical model with NHANES III to generate state-level prevalence estimates of adult overweight (11). Li et al used Massachusetts Behavioral Risk Factor Surveillance System (BRFSS) data to generate multilevel model-based zip code-level estimates to prioritize communities for obesity prevention (10). More recently, Congdon extended this framework for multilevel small-area estimation modeling by using BRFSS data with county-level covariates and predicted heart disease prevalence estimates for zip code tabulation area levels (13). We used a similar approach in this study to construct a multilevel model with county- and zip code-level covariates using NSCH 2007; we then predicted census block-group level small-area estimates (SAEs) of childhood obesity by combining the estimated model parameters and block-group level covariates with population counts for children, by age, sex, and race/ethnicity.

The objectives of our study were to 1) identify and evaluate individual and geographic factors that influence childhood obesity; 2) use multilevel small-area estimation methods to generate cost-effective data on the prevalence of childhood obesity at the block-group level for the United States; and 3) characterize the geographic disparities in childhood obesity by block groups, counties, and states.

## Methods

### Study population

The 2007 NSCH, a household landline–telephone-based interview survey stratified by state, has 91,642 completed interviews for children aged 0 to 17 years, with a minimum of 1,700 per state. The 2003 NSCH suggested that parent- or guardian-reported weight and height for children aged 0-9 years were not valid (17). We therefore included in this study only the 44,906 children aged 10 to 17 years with a validated obesity outcome; these children were from 2,618 counties and 13,291 zip codes in 50 states and the District of Columbia. The sample sizes for states range from 736 (Nevada) to 947 (North Dakota) with a median of 876 (Vermont) and a mean of 865. This geographic diversity in the sampled local communities (zip codes and counties) provides a solid basis on which to evaluate geographic effects on childhood obesity.

### Individual data

We obtained individual data on the study children’s age, sex, race/ethnicity, and obesity status from the 2007 NSCH. A child was considered obese if his or her body mass index (kg/m^2^) was equal to or greater than the sex- and age-specific 95th percentile on the Centers for Disease Control and Prevention (CDC) 2000 growth charts (18). Age was categorized into 2 groups (10–14 y,^15^–^17^ y) to match the age groups in block-group population data. Racial/ethnic categories were white, black, Asian, Hispanic, American Indian/Alaska Native, Native Hawaiian/Pacific Islander, multiracial, and other race.

### Geographic area data

Geographic area variables were block group, zip code, and county. We obtained the population count for children by sex, age, and race/ethnicity at the block-group level from 2010 ESRI Demographics (ESRI, Redlands, California). We also obtained median household income from ESRI for block groups, zip code areas, and counties, and we divided income into 8 levels. Lifestyle and urbanization levels (by block-group and zip code) were obtained from the 2010 ESRI Tapestry Segmentation dataset (19). The ESRI segmentation methodology incorporates sociodemographic, geographic, and physical features (eg, population density, city size, metropolitan status, proximity to economic and social centers) into community lifestyle and urbanization classifications. We used a 2006 National Center for Health Statistics (NCHS) 6-level urban-rural classification scheme for counties (20).

### Multilevel model development and estimation

Multilevel logistic regression models were constructed to evaluate the influence of individual child covariates and area-level covariates on a child’s obesity status in the NSCH. The full multilevel model was as follows:

NSCH child obesity status (yes or no) = sex + age + race/ethnicity (individual level) + median household income + lifestyle classifications + urbanization levels (zip-code level) + median household income + urban-rural (county-level) + random effects (state- and county-levels)

Zip-code level and county-level measures were included because neighborhood social and built environments and residential area (rural or urban) have been significantly associated with childhood obesity (21,22). We included zip code median household income and lifestyle classifications and urbanization levels to quantify local effects on childhood obesity. County median household income and urban-rural status were included to assess a regional effect on childhood obesity beyond neighborhood. State-level random effects represent the statewide social, economic, and political influences on childhood obesity. The multilevel logistic models were implemented in SAS 9.2 GLIMMIX (SAS Institute, Cary, North Carolina) by using maximum likelihood with the Laplace approximation estimation method.

To generate SAEs of childhood obesity prevalence at the block-group level, we estimated the obesity risk for a child calculated on the basis of age group, sex, and race/ethnicity for each block group from the 2010 ESRI Demographics data (19) by using the following predictive model:

A child’s predicted obesity risk = sex + age + race/ethnicity (individual-level) + median household income + lifestyle classifications + urbanization levels (block-group level) + median household income + urban-rural (county-level) + random effects (state-level)

The regression coefficients of block-group level covariates were adopted from those at the zip code level with an assumption that the block group and zip code-level influences on childhood obesity are at similar scales. Thus, each subpopulation of children defined by sex, age group, and race/ethnicity in each block group has its own obesity risk. The predicted number of obese children in each block group can be estimated by multiplying the predicted obesity risk by the number of children in the subpopulation. The overall model-based childhood obesity SAE in a block group is the population-weighted average of the sex-, age-, and racial/ethnic-specific SAEs for all the subpopulation groups within it. A Monte Carlo simulation approach was used to generate 95% confidence intervals (CIs) for all block group-predicted prevalence estimates of childhood obesity (23). The simulation was based on regression coefficients and their standard errors from the prevalence model on the basis of NSCH survey data, and 1,000 childhood obesity-prevalence SAEs were generated for each age-, sex-, and racial/ethnic-specific population for each block group.

### Evaluation of model-based SAEs

Direct survey estimates, such as those from NSCH, are often treated as the benchmark to evaluate and compare with model-based SAEs, to identify potential bias of model-based estimates, and to evaluate model specification (24). Although it would be ideal to compare SAEs at the block-group level, this comparison was not possible because NSCH cannot generate reliable block-group level estimates directly. However, we aggregated block-group level SAEs to county, state, and national levels and then compared them with NSCH direct survey estimates.

We evaluated model specification in 3 ways. First, we compared national-level direct survey and model-based estimates of childhood obesity prevalence for each age, sex, and race/ethnicity group to assess consistency between them. Second, we compared 40 state-specific model-based estimates with direct survey estimates available from both NSCH and YRBS because these surveys were designed to provide reliable state-level childhood obesity prevalence. Finally, we compared county-level, model-based SAEs with direct survey estimates for counties with data on at least 30 children and for which the ratio of standard errors to means was less than 0.3 (a reliability measure of survey estimation commonly used by the Centers for Disease Control and Prevention [CDC]) (25); we compared the estimates by using paired *t*-tests.

## Results

### Multilevel model adequacy and selection

We fitted 4 different multilevel logistic models with 1) state random effects, 2) county random effects, 3) both state and county random effects, and 4) zip-code random effects. No generally accepted criterion exists to evaluate the adequacy of multilevel models for small area estimation. We followed the recommendation that a multilevel model should explain at least 40% between area-level variance for the outcome measure of interest to justify model adequacy (26). Compared with their null models, the 4 full models in this study explained 59.8% (state random effect model) to 93.1% (zip code random effect model) area-level variances associated with childhood obesity (Table 1.).

**Table 1:** Proportion of Area-Level Variance for Childhood Obesity in Null Models Explained by Fixed Effects in Full Multilevel Models, by Type of Geographic Area

Logistic Model Level	Random Effects	Between-Area Variance (Null Model)^a^	SE	Between Area Variance (Full Model)^b^	SE	% Area-Level Variance Explained
I	Zip code	0.1950	0.03	0.0134	0.02	93.1
II	State	0.0530	0.01	0.0213	0.01	59.8
III	County	0.0928	0.02	0.0160	0.01	82.8
IV	State	0.0526	0.01	0.0211	0.01	60.0
	County (state)^c^	0.0456	0.01	0.0061	0.01	86.5

Abbreviation: SE, standard error.

a Null models are the models with random effects only.

b Full models are the models with random effects as well as fixed effects including age, sex, race/ethnicity, zip code level median household income, lifestyle and urbanity, and county level urban-rural status and median household income.

c Model IV has 2 random effects, state and county, which are nested in state.

For the full models, variance estimates at both zip code and county levels were not significant; only state-level variance estimates were significant (Table 1). We selected the full model with both state and county random effects having the smallest Akaike information criterion (27) for our small-area estimation.

We analyzed the details of the variables and the signs and significances of their regression coefficients in the full multilevel model with state- and county-level random effects (Table 2). After controlling for individual age, sex, race/ethnicity, and zip code-level median household income and lifestyle, we found that county-level median household income and rural–urban status were not significantly associated with childhood obesity. Zip-code urbanization levels were not significant. Therefore, county-level variables and zip code urbanization levels were excluded in the final multilevel model to predict risk for childhood obesity in a neighborhood.

**Table 2:** Regression Coefficients of Model Covariates for Estimating Predicted Risk for Childhood Obesity

Effect	Coefficients	SE	*P* Value
**Variable**	−3.4529	0.15	<.001
**Age, y**			
10-14	0.4117	0.03	<.001
15-17	Reference		
**Sex**			
Male	0.5753	0.03	<.001
Female	Reference		
**Race**			
White	Reference		
Black	0.6804	0.05478	<.001
Hispanic	0.5697	0.05	<.001
Asian	−0.1188	0.1714	.49
American Indian/Alaska Native	0.7070	0.14	<.001
Hawaii Native/Pacific Islander	0.7325	0.25	.003
Multiracial	0.1655	0.12	.16
Other races	0.0220	0.12	.85
**Zip code, median household income, $**			
1st octile (≤36,388)	0.8478	0.11	<.001
2nd octile (36,389–42,730)	0.7535	0.11	<.001
3rd octile (42,731–47,927)	0.6750	0.10	<.001
4th octile (47,928–53,351)	0.7204	0.10	<.001
5th octile (53,352–60,371)	0.5944	0.10	<.001
6th octile (60,372–68,513)	0.4319	0.08	<.001
7th octile (68,514–81,395)	0.3410	0.08	<.001
8th octile (≥81,396)	Reference		
**Zip code, lifestyles (** 19 **)**			
High Society	0.3810	0.12	.002
Upscale Avenues	0.3063	0.12	.01
Metropolis	0.3515	0.12	.005
Solo Acts	Reference		
Senior Styles	0.2716	0.13	.03
Scholars and Patriots	−0.1758	0.17	.29
High Hopes	0.3312	0.11	.003
Global Roots	0.4404	0.11	<.0001
Family Portrait	0.4092	0.12	.001
Traditional Living	0.3541	0.11	.001
Factories and Farms	0.4768	0.13	<.001
American Quilt	0.3711	0.13	.004
**Zip code, urbanization^a^**			
Principal Urban Centers I	Reference		
Principal Urban Centers II	0.0574	0.12	.63
Metro Cities I	−0.0739	0.11	.51
Metro Cities II	0.1325	0.11	.21
Urban Outskirts I	0.0561	0.11	.61
Urban Outskirts II	0.1739	0.13	.18
Suburban Periphery I	−0.0788	0.11	.49
Suburban Periphery II	0.0746	0.12	.53
Small Towns	0.1453	0.14	.29
Rural I	0.0458	0.13	.72
Rural II	0.2242	0.14	.11
**County, median annual household income, $**			
1st (≤40,647)	−0.1670	0.12	.15
2nd (40,648–46,323)	−0.1919	0.11	.08
3rd (46,324–51,075)	−0.1783	0.10	.09
4th (51,076–54,755)	−0.1616	0.10	.12
5th (54,756–57,898)	−0.0259	0.10	.81
6th (57,890–63,046)	−0.0455	0.10	.64
7th (63,047–76,686)	0.0031	0.09	.97
8th octile (≥76,687)	Reference		
**County National Survey of Children’s Health rural-urban status (** 20 **)**			
Central metro	Reference		
Fringe metro	0.0027	0.07	.97
Medium metro	0.0118	0.06	.83
Small metro	−0.0168	0.07	.80
Micropolitan	−0.0136	0.07	.84
Noncore rural	−0.0526	0.08	.51

a Urbanization groupings are from ESRI (19).

### Comparison between model-based and direct survey estimates

The national model-based childhood obesity estimate of 16.8% obesity among children aged 10 to 17 years was a nonsignificant 0.4 percentage points higher than the estimate based on direct survey (16.4%, <2.5% difference). At the state level, the observed childhood obesity prevalence ranged from 9.6% (Oregon) to 21.9% (Mississippi). Compared with these direct state-level estimates, the model-based estimates for each state fell within the 95% confidence intervals (CIs). The differences between state-specific direct survey estimates and model-based estimates ranged from –1.48 percentage points (West Virginia) to 1.73 percentage points (District of Columbia) with a median of 0.16 percentage points (Georgia). Paired *t*-tests showed no significant difference between direct-survey and model-based estimates. Finally, when we compared state-level model-based estimates for children aged 15 to 17 years with the observed prevalence of obesity found by YRBS for schoolchildren in grades 9 through 12, the average model-based SAEs of obesity prevalence for states with YRBS estimates was 12.6% compared with 12.2% for YRBS. A paired *t* test showed no significant difference between these 2 sets of estimates.

**Figure 1:**
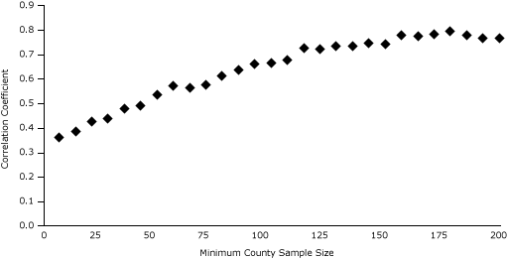
The relationship between correlation coefficients of model-based and direct survey estimates and minimum county sample size. The figure shows an increasing positive relationship between the correlation coefficients of model-based and direct survey estimates and the minimum county sample sizes.  As the minimum county sample size increases, the correlation between model-based and direct survey estimates also increases.  The correlation reaches approximately 0.90 and levels off once the minimum county sample size reaches 150.

At the county level, the model-based estimates are consistent with direct survey estimates. We plotted the relationship between model-based estimates and direct survey estimates for counties with a minimum sample size of 15. When the minimum sample size is exceeded for county-level direct survey estimates, the correlation between model-based and direct survey estimates increases substantially. When the minimum sample size nears 100, the correlation coefficients between model-based and direct survey estimates are consistently 0.7 or greater (Figure 1). By using our data suppression rules, we obtained 103 reliable county-level, direct-observed NSCH prevalence estimates. Although 12 model-based predicted SAEs were significantly higher or lower than their direct survey estimates, the median difference in county levels between model-based SAEs and direct-observed NSCH estimates was near zero (<0.0045 percentage points), and the first and third quartile differences were 1.38 and −1.98 percentage points, respectively.

**Table:** 

Minimum County Sample Size	Correlation Coefficient
15	0.36006
20	0.38515
25	0.42713
30	0.43893
35	0.47809
40	0.49237
45	0.53382
50	0.57392
55	0.56295
60	0.57453
65	0.61358
70	0.63623
75	0.66335
80	0.66428
85	0.67865
90	0.7267
95	0.72228
100	0.73423
110	0.73482
120	0.74902
130	0.74292
140	0.78155
150	0.77709
160	0.7826
170	0.79612
180	0.77936
190	0.76577
200	0.76577

### SAEs of childhood obesity prevalence from predictive model

We calculated the summary statistics of the confidence intervals (95% CIs) and coefficient of variation (CV) of model-based childhood obesity estimates at block-group, county, and state levels (Table 3). The 95% CIs for the block-group level have a large range (0.80%–20.0%), but their median is 2.02% and mean is 2.12% (Table 3). For block groups with large numbers of children, the 95% CIs are expected to be smaller. The CVs for block-group estimates have a range of 0.07% to 0.58%, a median of 0.14%, and a mean of 0.14%. Therefore, in most cases, model-based block-group estimates may be appropriate for ranking childhood obesity prevalence among communities. Model-based county and state SAEs are reliable.

**Table 3:** Summary Statistics of 95% Confidence Intervals and Coefficient of Variation of Childhood Obesity Estimates at Block Group, County, and State Levels

Geographic Unit	N	Minimum	1st Quartile	Median	3rd Quartile	Maximum	Mean	Interquartile Range
**95% CI**								
Block group	216,206	0.80	3.57	4.57	5.66	20.04	4.71	2.09
County	3,142	0.09	0.78	1.27	1.89	7.70	1.47	1.10
State	51^a^	0.04	0.07	0.10	0.17	0.44	0.13	0.10
**Coefficient of variation**								
Block group	216,206	0.07	0.12	0.14	0.16	0.58	0.14	0.04
County	3,142	<0.01	0.02	0.03	0.05	0.19	0.04	0.03
State	51	<0.01	<0.01	<0.01	0.01	0.01	<0.01	<0.01

Abbreviation: CI, confidence interval.

^a^ District of Columbia included in state count.

**Figure 2:**
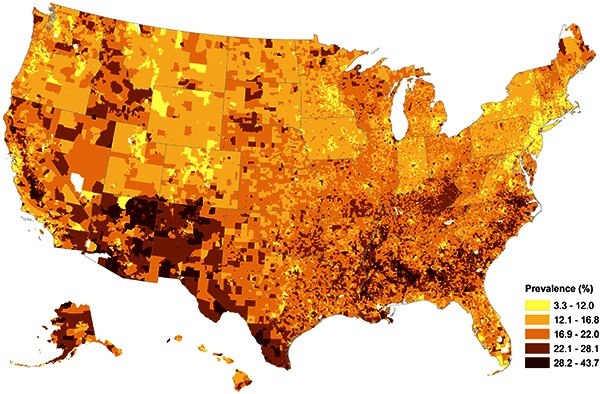
Model-Based Childhood Obesity Prevalence Estimates by Block Group in the United States, 2010.

The model-based national childhood obesity prevalence estimate was 16.8% on the basis of the 2010 ESRI Demographics population aged 10 to 17 years. The model-based SAEs of prevalence of childhood obesity ranged from 10.2% (Oregon) to 21.8% (District of Columbia) with a median of 14.9% at state level; from 7.2% to 31.9% with a median of 18.4% at the county level; and from 3.3% to 43.7% with a median of 16.8% at the block-group level. The overall geographic patterns of SAEs at the block-group level (Figure 2) show that obesity prevalence was higher in 1) large metropolitan areas such as New York, Los Angeles, and Chicago; 2) Southeastern and Midwestern rural areas; 3) along the US-Mexican border in Texas and California; and 4) in some local tribal areas in western and northern states.

## Discussion

We applied a multilevel modeling framework that incorporated demographic and geographic influences to estimate childhood obesity prevalence at local levels across the United States. Although SAEs of obesity prevalence among adults have been studied (10–12), to our knowledge, this is the first study of SAEs of obesity prevalence among children.

### The use of model-based SAEs in childhood obesity

Multilevel statistical modeling is an alternative approach to generating reliable SAEs; this model combines NSCH data with data from NCHS and other sources. But the estimates derived from the model must be used with caution for 2 reasons: first, the model-based estimates are the expected prevalence of childhood obesity for block groups given the demographic characteristics of the children in the population and their community’s socioeconomic status and lifestyle. These estimates are not; therefore, they could be very different from the actual prevalence of childhood obesity in a community. Second, we are not able to validate block-group level estimates, either internally or externally. In some cases, these estimates could be significantly biased.

### National health surveys for SAEs in childhood obesity prevalence

Although the sample populations of national health surveys usually are adequately representative demographically, they often lack sufficient geographic diversity to evaluate geographic effects on childhood obesity, especially for SAEs. NHANES aims to provide reliable national measures of obesity prevalence; however, it has limited geographic coverage because little more than 1.0% of 3,141 US counties are sampled (28). The BRFSS sampled all states and most counties, and its data have been used to generate county-level and zip-code level obesity prevalence for adults via model-SAE methods (10,12). However, BRFSS, like the National Health Interview Survey, has no obesity estimates for children. YRBS, a school-based survey, does have student-reported obesity measures but focuses on adolescents in grades 9 through 12; the schools surveyed are located in only 2% of US counties in 40 states, and the survey does not include obesity data for some large states such as California. Thus, the YRBS sampling framework has limited demographic and geographic coverage of children. We chose NSCH data for our analysis because it offers geographic diversity similar to that of BRFSS and validated obesity measures for the sampled children aged 10 to 17 years.

### Limitations

Our study had limitations. First, our study assumed that neighborhood influences on childhood obesity were similar at zip-code and block-group levels. On average, block groups are smaller geographic units than zip code areas, and this difference could cause some inference bias associated with the effects of modifiable area unit problems on the relationship between childhood obesity and neighborhood characteristics (29). The variance between areas increases when area size decreases (from state, county, to zip code) (Table 1). So we could expect greater variation in childhood obesity prevalence between block groups. We are examining this cross-level inference bias in a further study. Second, the NSCH 2007 relied on height and weight measurements reported by a parent or guardian. Parent-reported measures and directly measured height and weight for children yielded similar obesity estimates for children aged 9 to 11 years (30). No study has assessed the parent-reported bias for children aged 12 to 17 years. NSCH estimates of obesity prevalence for children aged 14 to 17 did not differ significantly from the YRBS estimates based on self-reported height and weights among adolescents in grades 9 through 12; this finding suggests that the bias due to parental report may not be significantly different from bias due to self-reports by adolescents. Another potential limitation is that childhood obesity may be associated with more neighborhood factors than just those in our multilevel model. Neighborhood grocery stores and restaurants and safety issues related to physical activity may also contribute to childhood obesity. Future multilevel SAE models of childhood obesity should take these community factors into account.

### Conclusion

Our study results show the effects of applying a multilevel, small-area modeling framework to NSCH data when zip code and county identifiers are used to estimate the prevalence of childhood obesity at the block-group level. The disparities among block groups, counties, and states show that “place of residence” may be as significant a contributor to the obesity epidemic among children as are individual characteristics such as race/ethnicity. Our estimates are useful for local public health programs when they set priorities and establish intervention goals (10). Health care systems and school- and community-based intervention programs to prevent childhood obesity will be more effective and efficient if they consider that local geographic factors contribute to local rates of obesity and need to be taken into account when intervention programs to reduce obesity are being designed. Model-based, small-area estimates of a population’s health status could be an important tool of public health assessment and surveillance (16).
